# Associations between static and dynamic changes of platelet counts and in-hospital mortality in critical patients with acute heart failure

**DOI:** 10.1038/s41598-024-59892-w

**Published:** 2024-04-21

**Authors:** Lili Wang, Tao Liu, Zhijian Zhu, Bing Wang, Zhigang Lu, Yesheng Pan, Lifang Sun

**Affiliations:** 1 Department of Cardiology, The Second Affiliated Hospital of Xuzhou Medical University, Xuzhou, China; 2 Department of Cardiology, Jinshan District Central Hospital Affiliated to Shanghai University of Medicine and Health Sciences, Shanghai, China; 3 Department of Cardiology, Shanghai Jiao Tong University Affiliated Sixth People’s Hospital, Shanghai, China

**Keywords:** Acute heart failure, Platelet count, In-hospital mortality, Change, Generalized additive mixed model, Medical research, Risk factors

## Abstract

To investigate the predictive value of baseline platelet count and its short-term dynamic changes in the prognosis of patients with acute heart failure (AHF) in the intensive care unit. Patients diagnosed with AHF in the medical information mart for intensive care III and their clinical data were retrospectively filtered. Patients were divided into survivor and non-survivor groups based on their prognosis during hospitalization, and differences in baseline data between groups were compared. Logistic regression models and restricted cubic spline (RCS) plots were performed to evaluate the relationship between baseline platelet counts and in-hospital mortality. Changes and trends in platelet counts were compared between the survivor and non-survivor groups after adjusting for confounders with the generalized additive mixing model (GAMM). A total of 2930 critical patients with acute heart failure were included, of which 2720 were survivors and 210 were non-survivors. Multiple logistic regression models revealed that baseline platelet count was an independent factor in hospital mortality (OR 0.997, 95% CI 0.994–0.999, *P*-value = 0.018). The RCS plot demonstrated a U-shaped dose–response relationship between baseline platelet count and in-hospital mortality. GAMM analysis suggested that the platelet counts decreased and then increased in the survivor group and gradually decreased in the non-survivor group, with a gradual increase of difference between two groups. After adjusting for confounders, the mean daily increase was −6.014 (95% CI −7.076–4.953, *P*-value < 0.001). Baseline platelet demonstrated a U-shaped dose–response relationship with adverse outcomes in critical patients with AHF. Early elevation of platelet was correlated with higher in-hospital mortality, indicating that tracking early changes in platelet might help determine the short-term prognosis of critical patients with AHF.

## Introduction

Heart failure (HF) affects about 26 million people worldwide, with an overall prevalence of approximately 1.5–4.0% and increasing, which place enormous pressure on healthcare systems^1–4^. Acute heart failure (AHF) is a syndrome defined as the new onset or worsening of symptoms and signs of HF, mostly related to high mortality^5^. Hence, accurate assessment of severity in AHF patients have very important significance of improve prognosis and reduce medical burden. However, the complexity and heterogeneity of pathophysiology contribute to the severity of clinical symptoms in AHF patients ranging from mild to lethal^6^. Therefore, accurate assessment of disease severity in AHF patients has become a challenge.

Platelets (PLT), a component of blood, are involved in coagulation, the immune response and inflammation, and it has been proven to be closely related to the prognosis of critically ill patients^7,8^. Wang et al. study found that as a strong predictor of potential risk of death of critical patient with sepsis, every 10 × 109/L increase in platelets was associated with a 13% decrease in risk of the death^9^. In addition, the platelet to lymphocyte ratio (PLR) may be useful in supplementary assessment of short-term mortality risk in AHF patients^10^. However, few studies have explored the relationship between PLT and in-hospital death among critical patients with AHF. Furthermore, dynamical PLT change is a complementary predictor to baseline PLT for critical patient survival, even in patients without thrombocytopenia^11^. Therefore, dynamic assessment of platelet changes may be more conducive to an effective assessment of the overall condition and severity of AHF patients.

In the present study, we analyzed data from the medical information mart for intensive care III (the MIMIC-III) database, and to further revealed relationship of both baseline PLT and early changes in PLT within the first week after admission on in-hospital mortality among critical AHF patients.

## Materials and methods

### Source of data

All data of this study were obtained from the Medical Information Mart for Intensive Care III (MIMIC-III) database (version 1.4). The MIMIC-III database records medical data on patients in the intensive care unit (ICU) at Beth Israel Deaconess Medical Center between 2001 and 2012, with a collection of 53,423 ICU admissions, including a large amount of physiological data and medical records^12^. All data are authentic and publicly accessible for free. No informed consent was required from the patients as their basic personal information was anonymized (https://mimic.physionet.org/). Data for the present study was extracted by one author, Tao Liu, passed the training test and obtained permission to download and use the database (ID: 9008147).

### study population

We initially recruited 3614 critical patients with the diagnosis of AHF from the MIMIC-III database, of which 607 participants were excluded due to repeated hospitalization and 42 participants were excluded due to death within 3 days of admission. Of the remaining 2,965 participants, 35 participants were excluded due to missing data. Ultimately, 2930 critical patients with AHF were identified for analysis, including 2720 survivors and 210 non-survivors.

### Data extraction

The involved data was extracted with PostgreSQL tool. Demographic information: gender, age, ethnicity, and marital status; Vital signs: heart rate (HR), respiratory rate (RR), systolic blood pressure (SBP), diastolic blood pressure (DBP), pulse oxygen saturation (SpO_2_), and temperature; 24 h urine output; Indicators of laboratory tests: platelet, partial pressure of oxygen (PaO_2_), partial pressure of carbon dioxide (PaCO_2_), potential of hydrogen (pH), N-Terminal pro-brain natriuretic peptide (NT-proBNP), white blood cell (WBC), neutrophile (N), lymphocyte (L), C reactive protein (CRP), and estimated glomerular filtration rate (eGFR); Comorbidities: sepsis, stroke, pneumonia, acute myocardial infarction (AMI), atrial fibrillation (AF), liver disease, chronic kidney disease (CKD), chronic obstructive pulmonary disease (COPD), respiratory failure, and cancer; Therapeutic measures: renal replacement therapy (RRT), ventilation, and vasoactive drugs; Scoring scales for ICU disease: the Sequential Organ Failure Assessment (SOFA) Score and the Simplified Acute Physiology Score II (SAPS II). Length of hospital: length of ICU stay and length of hospital stay. All of the above baseline values were the first measured within the first 24-h after admission to the ICU. In addition, the registration information of admission and discharge was recorded, including the length of hospitalization, time of death, and length of stay in the ICU.

### Outcomes and main exposure variables

The outcome of this study was in-hospital mortality, which was death from all causes during the patient's hospitalization. The independent variables were baseline platelet count and dynamically changed platelet count. Dynamically changed platelet counts were platelet counts measured daily during the one week stay in the ICU. The interval time between repeated measurements of platelet counts was irregular.

### Statistical analysis

All data were analyzed with R Studio software (Version 4.2.1). Continuous variables that conformed to normal distribution were expressed as mean (standard deviation) and the t-tests were applied; Continuous variables not satisfying normal distribution were shown as median (interquartile spacing) [M (Q1, Q3)] and the Wilcoxon rank-sum tests were carried out; Categorical variables were presented as percentages (%) and the *X*^2^ tests were conducted. Sequentially, logistic regression models were constructed to assess the association of baseline platelet count and baseline tertile platelet count with in-hospital mortality in critical patients with AHF, and the results were displayed in the form of odds ratios (ORs) and 95% confidence intervals (CIs). Model 1: unadjusted variables; Model 2: Adjusted for age, gender, ethnicity, and marital status; Model 3: Adjusted for age, ethnicity, HR, RR, SBP, DBP, temperature, PO_2_, PCO_2_, pH, 24-h urine output, sepsis, AMI, AF, respiratory failure, RRT, ventilation, vasoactive drugs, SOFA, SASP II, NT-proBNP, WBC, N, L, CRP, eGFR, length of ICU stay, and length of hospital stay. Meanwhile, the relationship between baseline platelet counts and in-hospital mortality was re-analyzed utilizing stepwise regression to avoid multicollinearity among the covariates (Supplementary Table S1). The nonlinear relationship between baseline platelet count levels and in-hospital mortality were explored by adjusting the multivariate restricted cubic spline (RCS) regression model. The inflection point was calculated based on the recursive algorithm, and then the threshold effect analyses were conducted by the segmented logistic regression model (Supplementary Table S3). In addition, we conducted ROC for PLT, eGFR, and NT-proBNP on predicting in-hospital mortality, and we also examine correlation analysis of in-hospital mortality with WBC, CRP, neutrophil, lymphocyte, and PLT.

Differences in dynamical platelet counts between survivors and non-survivors during the first week after ICU admission were compared (Supplementary Table S4). Finally, generalized additive mixed models (GAMM) were constructed to investigate the relationship between dynamic platelet counts in the first week and in-hospital mortality in critical patients with AHF admitted to ICU. GAMM is usually performed to the analyse data obtained repeatedly, especially when the data have missing values or are duplicated irregularly^13,14^. *P*-value < 0.05 was considered to be statistically significant.

### Ethics statement

Data of the present study was from the MIMIC-III database. The MIMIC III database was approved to build by the Institutional Review Boards of Beth Israel Deaconess Medical Center (Boston, MA) and the Massachusetts Institute of Technology (Cambridge, MA). Data for the present study was extracted by one author, Tao Liu, who has completed the online training course and passed the exam (ID: 9008147).

## Results

### Baseline characteristics of the study population

A total of 2930 critical patients with AHF were included with a mean age of 72.83 (13.14) years, of which 1486 (50.7%) were males and 1444 (49.3%) were females. After tracking hospitalization information, 210 (7.17%) deaths were recorded. Compared with the non-survivor group, patients in the survivor group were younger, had a higher proportion of Whites and Blacks, higher levels of temperature, DBP, SBP, PO_2_, PCO_2_, pH, eGFR, and platelets, lower levels of HR, RR, SAPSS II scores, and SOFA scores, and NT-proBNP, WBC, L, N, CRP, length of ICU stay, length of hospital stay, and lower prevalences of sepsis, pneumonia, AMI, AF, and respiratory failure, as well as lower proportions of ventilation, RRT and vasoactive medication use (all *P*-value < 0.05). There were no statistically significant differences between the two groups in terms of the proportion of gender and marital status, levels of SPO_2_, prevalence of stroke, COPD, cancer, hypertension, diabetes and CKD (all *P*-value > 0.05) (Table 1). In addition, the results of propensity score matching (1:3) was replaced in Supplementary Table S2.

**Table 1:** Baseline characteristics of study participants.

Variables	Overall	Survivor group	No-survivor group	*P*-value
	N = 2930	N = 2720	N = 210	
Age, years	72.83 (13.14)	72.51 (13.21)	76.93 (11.57)	< 0.001
Male, n (%)	1486 (50.7)	1379 (50.7)	107 (51.0)	> 0.99
Ethnicity, n (%)				0.017
White	2199 (75.1)	2042 (75.1)	157 (74.8)	
Black	311 (10.6)	299 (11.0)	12 (5.7)	
Asian	92 (3.1)	85 (3.1)	7 (3.3)	
Other	328 (11.2)	294 (10.8)	34 (16.2)	
Married, n (%)	1344 (45.9)	1240 (45.6)	104 (49.5)	0.303
**Vital signs**				
Temperature, ℃	36.68 (0.56)	36.69 (0.55)	36.59 (0.67)	0.018
HR, time/minute	84.34 (15.49)	84.04 (15.32)	88.13 (17.22)	< 0.001
RR, time/minute	19.96 (3.81)	19.86 (3.74)	21.20 (4.39)	< 0.001
SBP, mmHg	115.94 (16.27)	116.50 (16.25)	108.79 (14.74)	< 0.001
DBP, mmHg	58.55 (10.16)	58.75 (10.17)	55.96 (9.77)	< 0.001
**Blood gas**				
PH	7.38 (0.09)	7.38 (0.09)	7.35 (0.11)	< 0.001
SpO2, %	96.74 (1.97)	96.74 (1.96)	96.74 (2.13)	0.96
PaO_2_, mmHg	123.00 (81.00, 225.40)	125.00 (82.00, 231.55)	99.00 (72.40, 173.50)	< 0.001
PaCO_2_, mmHg	42.00 (37.00, 49.00)	42.20 (37.00, 49.00)	41.00 (33.70, 48.00)	0.014
**Laboratory examination**				
NT-proBNP, pg/ml	8365.64 (5454.11, 10,750.68)	8198.54 (5367.85, 10,543.67)	10,872.15 (8037.24, 11,955.53)	< 0.001
WBC, (109/L)	9.70 (7.00, 13.30)	9.60 (6.95, 13.20)	10.40 (7.20, 14.70)	0.034
N, (109/L)	4.30 (3.41, 9.16)	4.25 (3.38, 9.02)	4.80 (3.88, 9.55)	< 0.001
L, (109/L)	2.51 (2.29, 2.77)	2.47 (2.27, 2.73)	2.99 (2.74, 3.23)	< 0.001
CRP, (mg/dL)	11.00 (2.20, 81.00)	11.00 (2.10, 81.04)	14.10 (4.00, 55.55)	0.018
eGFR, mL/min/1.73 m^2^	61.11 (37.78, 90.12)	62.56 (39.61, 91.37)	36.64 (25.92, 65.50)	
**Comorbidities**				
Sepsis, n (%)	357 (12.2)	291 (10.7)	66 (31.4)	< 0.001
Stroke, n (%)	99 (3.4)	87 (3.2)	12 (5.7)	0.081
Pneumonia, n (%)	962 (32.8)	846 (31.1)	116 (55.2)	< 0.001
AMI, n (%)	183 (6.2)	162 (6.0)	21 (10.0)	0.029
Liver disease, n (%)	4 (0.1)	4 (0.1)	0 (0.0)	> 0.99
COPD, n (%)	189 (6.5)	182 (6.7)	7 (3.3)	0.078
Cancer, n (%)	66 (2.3)	57 (2.1)	9 (4.3)	0.069
Hypertension, n (%)	1169 (39.9)	1083 (39.8)	86 (41.0)	0.802
DM, n (%)	1220 (41.6)	1143 (42.0)	77 (36.7)	0.149
AF, n (%)	1433 (48.9)	1312 (48.2)	121 (57.6)	0.011
CKD, n (%)	1092 (37.3)	1009 (37.1)	83 (39.5)	0.531
Respiratory failure, n (%)	884 (30.2)	758 (27.9)	126 (60.0)	< 0.001
Saps II score	38.00 (31.00, 46.00)	37.00 (30.00, 45.00)	50.00 (42.00, 58.00)	< 0.001
Sofa score	4.00 (2.00, 6.00)	4.00 (2.00, 6.00)	6.00 (4.00, 8.00)	< 0.001
24-h urine output, ml	1550.00 (922.00, 2338.75)	1593.50 (973.00, 2366.25)	1001.50 (516.25, 1657.76)	< 0.001
RRT, n (%)	243 (8.3)	204 (7.5)	39 (18.6)	< 0.001
Ventilation, n (%)	2545 (86.9)	2,353 (86.5)	192 (91.4)	0.044
Vasopressin, n (%)	1177 (40.2)	1032 (37.9)	145 (69.0)	< 0.001
Length of ICU stay, days	2.96 (1.63, 5.50)	2.83 (1.54, 5.08)	5.94 (3.46, 11.25)	< 0.001
Length of hospital stay, days	8.88 (5.71, 14.08)	8.77 (5.65, 13.88)	10.50 (6.63, 18.38)	< 0.001
Platelet counts, 10^9^/L	209.00 (158.00, 265.00)	211.00 (161.75, 266.00)	182.00 (119.00, 251.00)	< 0.001

HR, heart rate; RR, respiratory rate; SBP, systolic blood pressure; DBP, diastolic blood pressure; PH, potential of hydrogen; PaO_2_, partial pressure of oxygen; PaO_2,_ partial pressure of oxygen; PaCO_2,_ partial pressure of carbon dioxide; AMI, acute myocardial infarction; COPD, chronic obstructive pulmonary disease; DM, diabetes mellitus; AF, atrial fibrillation; CKD, chronic kidney diseases; SAPS II, simplified acute physiologic score II; SOFA score, sequential organ failure assessment; RRT, renal replacement therapy; NT-proBNP: N-Terminal pro-brain natriuretic peptide; WBC, white blood cell; N, neutrophile; L, lymphocyte; CRP, C reactive protein, and eGFR, estimated glomerular filtration rate.

### Association between in-hospital mortality and baseline platelet

The detailed results of the multivariate logistic regression are shown in Table 2**.** When considering platelets as a continuous variable, each 1 × 10^9^/L increase in platelet count corresponded to a OR (95% Cl) for in-hospital mortality of 0.997 (95% CI 0.994–0.999, *P*-value = 0.018). Similar results were found when stepwise regression regression analysis was performed (Supplementary Table S1. Baseline platelets were considered as a tertile variable, and the fully adjusted OR (95% CI) for in-hospital mortality was 0.548 (0.328–0.917, *P*-value = 0.022) and 0.712 (0.431–1.176, *P*-value = 0.185) for the Q2 and Q3 groups, respectively, on the basis of Group Q1 as a reference.

**Table 2:** Association between baseline platelet counts and the risk of hospital mortality among critical AHF patients.

Outcomes		Model 1			Model 2			Model 3		
		OR	95% CI	*P*-value	OR	95% CI	*P*-value	OR	95% CI	*P*-value
PLT Per 1 sd		0.995	(0.993, 0.997)	< 0.001	0.995	(0.993, 0.997)	< 0.001	0.997	(0.994, 0.999)	0.018
PLT tertiles	Case, N									
Q1	987	1.0 (Reference)			1.0 (Reference)			1.0 (Reference)		
Q2	969	0.462	(0.323, 0.652)	< 0.001	0.487	(0.339, 0.692)	< 0.001	0.548	(0.328, 0.917)	0.022
Q3	974	0.518	(0.367, 0.723)	< 0.001	0.543	(0.382, 0.764)	< 0.001	0.712	(0.431, 1.176)	0.185

Model 1: Unadjusted for any variables.

Model 2: Adjusted for age, gender, ethnicity, and married.

Model 3: Adjusted for age, ethnicity, HR, RR, SBP, DBP, temperature, PaO_2_, PaCO_2_, pH, 24-h urine output, AMI, AF, respiratory failure, RRT, ventilation, vasoactive drugs, SOFA, SASP II, NT-proBNP, WBC, N, L, CRP, eGFR, length of ICU stay, and length of hospital stay.

OR, odds ratio; CI, confidence interval; HR, heart rate; RR, respiratory rate; SBP, systolic blood pressure; DBP, diastolic blood pressure; PaO_2,_ partial pressure of oxygen; PaCO_2,_ partial pressure of carbon dioxide; pH, potential of hydrogen; AF, atrial fibrillation; AMI, acute myocardial infarction; RRT, renal replacement therapy; SOFA score, sequential organ failure assessment; SAPS II, simplified acute physiologic score II; NT-proBNP: N-Terminal pro-brain natriuretic peptide; WBC, white blood cell; N, neutrophile; L, lymphocyte; CRP, C reactive protein, and eGFR, estimated glomerular filtration rate.

### The non-linear relationship

**Figure 1:**
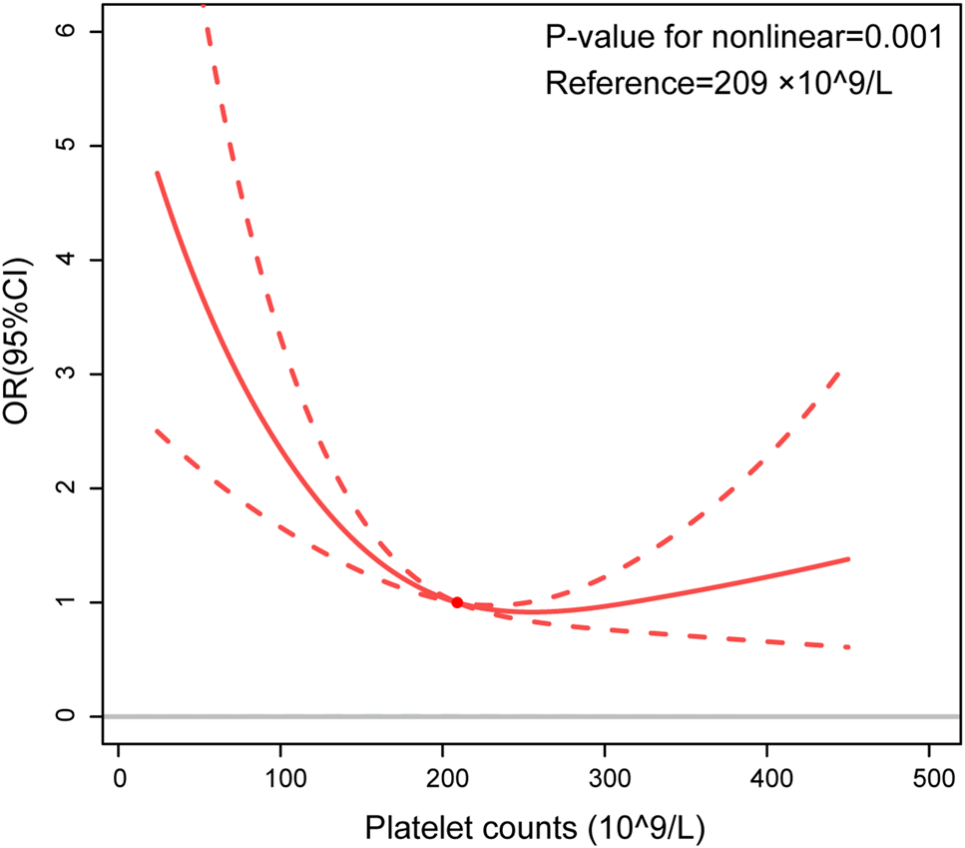
The RCS for association between baseline platelet counts and in-hospital mortality with adjusting for adjusted for age, ethnicity, HR, RR, SBP, DBP, temperature, PaO_2_, PaCO_2_, pH, 24-h urine output, AMI, AF, respiratory failure, RRT, ventilation, vasoactive drugs, SOFA, SASP II, NT-proBNP, WBC, N, L, CRP, eGFR, length of ICU stay, and length of hospital stay.

RCS regression model adjusted for model 3 showed that the relationship between baseline platelets and in-hospital mortality was U-shaped in critical patients with AHF (Fig. 1). The results of two piecewise linear regression revealed that the in-hospital mortality was lowest when the baseline platelet count was 209 × 10^9^/L (Supplementary Table S3). Below the inflection point, in-hospital mortality declined with increasing platelet counts [OR (95% CI): 0.990 (0.985–0.995), *P*-value < 0.001]. Above the inflection point, in-hospital mortality rose with increasing platelet counts [OR (95% CI): 1.002 (0.998–1.006), *P*-value = 0.380]. The C-statistics of platelet count were calculated. PLT, e-GFR and NT-proBNP all have certain clinical predictive value in predicting in-hospital death in patients with AHF, and PLT could improve predictive value of e-GFR and NT-proBNP in predicting in-hospital death (Supplementary Table S5). Meanwhile, we also analyzed correlation of between PLT, WBC, CRP, neutrophil, lymphocyte and hospital mortality. PLT are negatively related hospital mortality, and positively related to WBC, neutrophil. WBC, neutrophil, and lymphocyte are positively related to hospital mortality (Supplementary Figure S1).

### Association between in-hospital mortality and dynamically changed platelets

**Figure 2:**
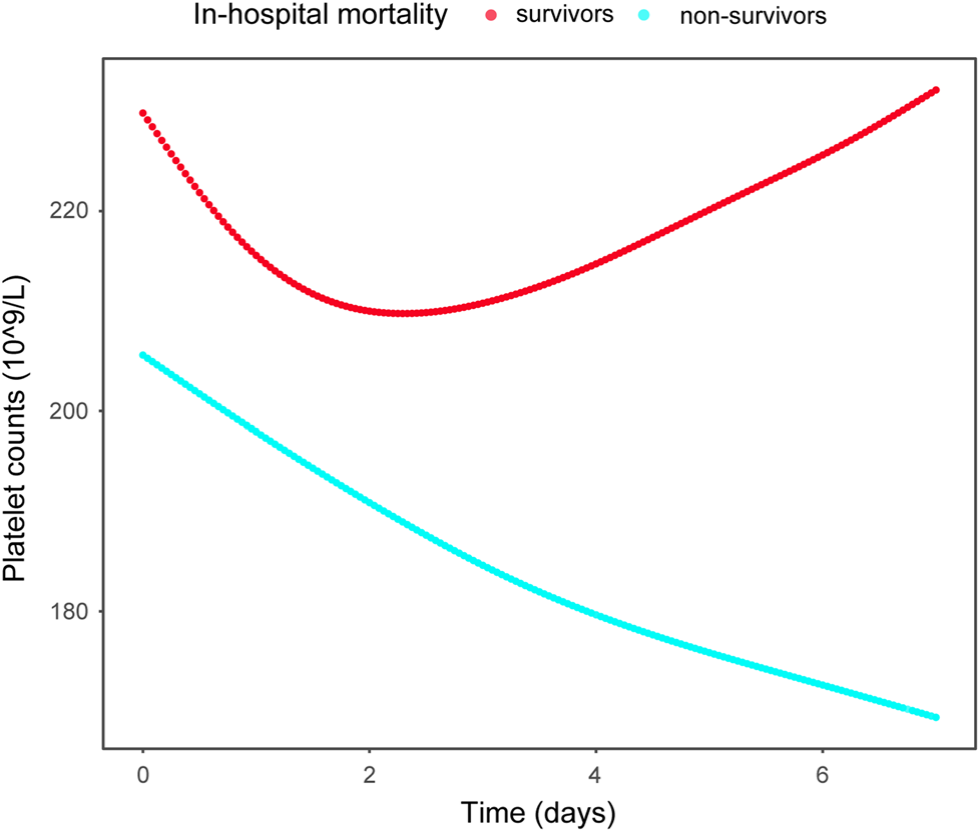
Evaluation of association between platelet counts (1–7 days) and in-hospital mortality in critical patients with AHF using generalized additive mix model (GAMM), and adjusting for adjusted for age, ethnicity, HR, RR, SBP, DBP, temperature, PaO_2_, PaCO_2_, pH, 24-h urine output, AMI, AF, respiratory failure, RRT, ventilation, vasoactive drugs, SOFA, SASP II, NT-proBNP, WBC, N, L, CRP, eGFR, length of ICU stay, and length of hospital stay.

We compared the dynamics of platelet counts at 4 time points: 1st, 2nd-3rd, 4th-5th, and 6th-7th days after ICU admission between the survivor and non-survivor groups (Supplementary Table S3). The difference in platelet counts between the two groups at each time point was statistically significant. In the next step, the trajectories of platelet count over time for two groups were plotted with the GAMM model fully adjusted for confounding factors (Fig. 2). Platelet counts of patients in the survivor group showed a decreasing and then increasing trend with a U-shape. The platelet counts in the non-survivor group decreased gradually within 1 week after admission to ICU. Overall, the difference in platelet counts between the two groups tended to increase over time, and platelet counts were consistently higher in the survivor group than in the non-survivor group. Platelet counts increased by a mean of −5.989 per day in the non-survivor group compared to the survivor group. Consistent results were also observed in Model 3 (β = −6.014, 95% CI −7.076–4.953, *P*-value < 0.001) (Table 3).

**Table 3:** To analyze association between changes (1–7 days) platelet counts and in-hospital mortality among critical AHF patients utilizing a generalized additive mixed model (GAMM).

In-hospital mortality	Model 1		Model 2		Model 3	
	β (95%CI)	*P*-value	β (95%CI)	*P*-value	β (95%CI)	*P*-value
Time	0.668 (0.357 to 0.979)	< 0.001	0.668 (0.357 to 0.979)	< 0.001	0.697 (0.387 to 1.008)	< 0.001
Death	−13.005 (−23.878 to 2.132)	0.019	−11.488 (−22.320 to 0.657)	0.038	−8.500 (−19.408 to 2.408)	0.127
Death × day	−5.989 (−7.051 to 4.927)	< 0.001	−5.987 (−7.048 to 4.924)	< 0.001	−6.014 (−7.076 to 4.953)	< 0.001

Time, the mean increasing of PLT daily in the survival group over time (1–7 days); Death, the mean difference of PLT at admission between survivor group and No-survivor group; Death × day, compared with survivor group, the mean increasing in PLT daily under the condition of No-survivor group.

Model 1: Unadjusted for any variables.

Model 2: Adjusted for age, gender, ethnicity, and married.

Model 3: Adjusted for age, ethnicity, HR, RR, SBP, DBP, temperature, PaO_2_, PaCO_2_, pH, 24-h urine output, AMI, AF, respiratory failure, RRT, ventilation, vasoactive drugs, SOFA, SASP II, NT-proBNP, WBC, N, L, CRP, eGFR, length of ICU stay, and length of hospital stay.

CI, confidence interval; HR, heart rate; RR, respiratory rate; SBP, systolic blood pressure; DBP, diastolic blood pressure; PaO_2,_ partial pressure of oxygen; PaCO_2,_ partial pressure of carbon dioxide; pH, potential of hydrogen; AF, atrial fibrillation; AMI, acute myocardial infarction; RRT, renal replacement therapy; SOFA score, sequential organ failure assessment; SAPS II, simplified acute physiologic score II; NT-proBNP: N-Terminal pro-brain natriuretic peptide; WBC, white blood cell; N, neutrophile; L, lymphocyte; CRP, C reactive protein, and eGFR, estimated glomerular filtration rate.

## Discussion

The present study observed the dynamic evolution of platelet counts in critical patients with AHF from the 1st to the 7th day after ICU admission in order to analyze the relationship between platelet counts and poor outcomes. A U-shaped association between baseline platelet count and in-hospital mortality was found. Platelet counts of patients in the survivor group presented a first decreasing and then increasing trend on days 1–7 after admission to the ICU, while the non-survivor group showed a continuous decreasing trend, and platelet counts in the survivor group were consistently higher than those in the non-survival group at the same time points. After sufficient adjustment for confounders, we found that the early decline in platelet counts was an independent predictor of in-hospital death in critical patients with AHF.

The association of decreased platelet count with poor prognosis has been well supported in studies among different populations, especially in ICU patients^15–18^. Nihat Polat et al. revealed that in patients with acute decompensated HF, baseline platelet counts were significantly higher in alive patients than in deceased patients, and lower platelet counts predicted an increased risk of death at one year^19^. Results from the MyoVasc study showed that lower platelet counts were associated with poorer cardiac function and were an independent predictor of worsening heart failure, with a median follow-up of 2.24 years^20^. The indicators in all three studies were platelet counts measured at a single time point at baseline and do not take into account the fact that platelet counts change dynamically in actual clinical management. Current studies on the relationship between platelet dynamic trajectories and prognosis have focused on severe burn patients, septic shock patients, and cancer patients, and studies involving critical patients with AHF have not been observed^21–23^. We investigated the relationship between early changes in platelet counts and the risk of in-hospital mortality in critical patients with AHF, which more rationally assesses the evolution of the disease holistically.

Univariate analysis of this study indicated that baseline platelet counts were higher in the alive group than in the dead group, which is consistent with previous studies^19,24^. The results of the multivariate logistic regression also endorsed these findings, regardless of considering platelets as a continuous variable or a tertile variable. Immediately after, GAMM was implemented to explore early changes in platelet counts in critically ill AHF patients and their relationship to the risk of in-hospital mortality. The results showed that the platelet counts of those who died were lower than those of the survivors on the 1st-7th days and showed a continuous decline, with a statistically significant difference between the groups. In summary, we need to focus on both the platelet count at baseline and the dynamic changes in platelet count on the patient's prognosis.

AHF is a clinical syndrome characterized by the rapid onset or sudden deterioration of symptoms or signs of HF with elevated plasma natriuretic peptide levels caused by multiple etiologies^25^. It is well known that inflammation plays an important role in the pathogenesis of HF, and platelets are the "instigators" and "'participants" of inflammation^26^. During the inflammatory response, vascular endothelial cells are damaged, followed by platelet adhesion, aggregation, and thrombus formation^27^. In addition, platelet activation promotes the inflammatory response and the process of atherosclerosis by initiating monocyte transformation, recruiting inflammatory cells, and promoting foam cell formation^28^. On the other hand, platelet activation also releases mediators such as serotonin, adenine nucleotides, and other mediators thereby activating protective cardiac pathways^29,30^. It has been reported that injecting autologous platelet gel into the myocardium of mice can improve cardiac function and delay ventricular remodeling after myocardial infarction^31^. Therefore, platelets have both protective and damaging effects on the heart. Platelets in patients with heart failure are usually overactivated, which may be attributed to hemodynamic changes and structural damage to the vascular system^29,32,33^. Platelet overconsumption and high destruction lead to a decrease in peripheral blood platelet counts. In addition, low platelet count is related to activation of the renin–angiotensin–aldosterone system ^34^. Therefore, the poorer prognosis among critical patients AHF with low platelet count might appear. Moreover, platelets play a role in the anti-infectious response^35^. Previous studies have shown that when the daily PLT count increases by 31 × 10^9^/L in ICU patients with baseline PLT counts exceeding 300 × 10^9^/L, the risk of death in ICU patients is reduced by 25%^11^. In addiion, the daily PLT count increases by 10 × 10^9^/L in ICU patients with baseline PLT counts below 100 × 10^9^/L, the risk of death in ICU patients with sepsis is reduced by 13%^9^. Therefore, a certain increase in PLT count leads to better prognosis, which is consistent with our findings. In the present study, we found a U-shaped dose–response association of between baseline PLT counts and in-hospital mortality in critical patients with AHF.

There are some limitations in this study: (1) Although the confounding factors were adequately adjusted, it was not possible to control all the confounding parameters that could affect the results. (2) The retrospective study design, which could only show the correlation between PLT counts and in-hospital mortality, did not prove causality. (3) This is a single-center study, and the results are representative of the study population, with some limitations on extrapolation. Therefore, further multi-center registries of large-scale prospective studies are needed to confirm. (4) This study did not conduct further subgroup analysis on the causes of heart failure. Later studies need to conduct subgroup analysis on the causes of heart failure to verify the stability of the relationship between PLT and in-hospital mortality. (5) This article focuses on correlations, and more research is needed to uncover the exact mechanism of the association between PLT counts and patients with AHF.

## Conclusions

There was a U-shaped relationship between baseline PLT counts and in-hospital mortality in critical patients with AHF. Early elevation of PLT was correlated with higher in-hospital mortality, indicating that tracking early changes in PLT might help determine the short-term prognosis of critical patients with AHF. In addition, PLT count provides certain clinical predictive value for the short-term prognosis of critical patients with AHF. Future research should focus on investigating the association between PLT counts and long-term outcomes.

### Supplementary Information

Supplementary Information.

## Data Availability

The data supporting the findings of the present paper could be found at https://mimic.mit.edu/.
