# Supplementary material for: Associations between static and dynamic changes of platelet counts and in-hospital mortality in critical patients with acute heart failure
Source: Sci Rep. 2024 Apr 21;14:9147. doi: 10.1038/s41598-024-59892-w (PMC11033279; doi:10.1038/s41598-024-59892-w)
Supplement: Supplementary file 1 — Supplementary Information. [file 41598_2024_59892_MOESM1_ESM.docx]

**Supplementary Table 1.** The relationship between platelet counts and in-hospital death among critical AHF patients using stepwise regression regression analysis.

| Model | OR | 95% CI | *P*-value |
| --- | --- | --- | --- |
| Model 1 | 0.995 | (0.993,0.997) | <0.001 |
| Model 2 | 0.995 | (0.993,0.997) | <0.001 |
| Model 3 | 0.996 | (0.994,0.999) | 0.004 |

Model 1: Unadjusted for any variables.

Model 2: Adjusted for age, gender, ethnicity, and married.

Model 3: Adjusted for age, ethnicity, HR, RR, SBP, DBP, temperature, PaO2, PaCO2, pH, 24-hour urine output, AMI, AF, respiratory failure, RRT, ventilation, vasoactive drugs, SOFA, SASP II, NT-proBNP, WBC, N, L, CRP, eGFR, length of ICU stay, and length of hospital stay.

Abbreviations: OR, odds ratio; CI, confidence interval; HR, heart rate; RR, respiratory rate; SBP, systolic blood pressure; DBP, diastolic blood pressure; PaO2, partial pressure of oxygen; PaCO2, partial pressure of carbon dioxide; pH, potential of hydrogen; AF, atrial fibrillation; AMI, acute myocardial infarction; RRT, renal replacement therapy; SOFA score, sequential organ failure assessment; SAPS II, simplified acute physiologic score II; NT-proBNP: N-Terminal pro-brain natriuretic peptide; WBC, white blood cell; N, neutrophile; L, lymphocyte; CRP, C reactive protein, and eGFR, estimated glomerular filtration rate.

**Supplementary Table 2.** Baseline characteristics of study participants based on propensity score matching (1:3).

| Variables | Overall | Survivor group | No-survivor group | *P*-value |
| --- | --- | --- | --- | --- |
|  | N = 840 | N = 630 | N = 210 |  |
| Age, years | 76.63 (11.49) | 76.52 (11.47) | 76.93 (11.57) | 0.655 |
| Male, n (%) | 423 (50.4) | 316 (50.2) | 107 (51.0) | 0.905 |
| Ethnicity, n (%) | |  |  | 0.132 |
| White | 631 (75.1) | 474 (75.2) | 157 (74.8) |  |
| Black | 68 (8.1) | 56 (8.9) | 12 (5.7) |  |
| Asian | 36 (4.3) | 29 (4.6) | 7 (3.3) |  |
| Other | 105 (12.5) | 71 (11.3) | 34 (16.2) |  |
| Married, n (%) | 369 (43.9) | 265 (42.1) | 104 (49.5) | 0.071 |
| **Vital signs** |  |  |  |  |
| Temperature, ℃ | 36.63 (0.62) | 36.64 (0.60) | 36.59 (0.67) | 0.362 |
| HR, time/minute | 85.91 (16.09) | 85.17 (15.64) | 88.13 (17.22) | 0.021 |
| RH, time/minute | 20.68 (4.05) | 20.51 (3.91) | 21.20 (4.39) | 0.032 |
| SBP, mmHg | 112.42 (15.35) | 113.63 (15.37) | 108.79 (14.74) | <0.001 |
| DBP, mmHg | 56.89 (9.60) | 57.19 (9.53) | 55.96 (9.77) | 0.106 |
| **Blood gas** |  |  |  |  |
| PH | 7.37 (0.09) | 7.37 (0.09) | 7.35 (0.11) | 0.007 |
| SpO2, % | 96.73 (2.02) | 96.72 (1.98) | 96.74 (2.13) | 0.888 |
| PaO_2_, mmHg | 107.00 [77.85, 180.05] | 110.00 [79.00, 185.10] | 99.00 [72.40, 173.50] | 0.035 |
| PaCO_2_, mmHg | 41.00 [35.00, 48.00] | 41.00 [35.40, 48.15] | 41.00 [33.70, 48.00] | 0.622 |
| **Laboratory examination** | |  |  |  |
| NT-proBNP, pg/ml | 10103.03 [7565.89, 11752.03] | 9848.13 [7525.83, 11611.96] | 10872.15 [8053.49, 11945.82] | 0.002 |
| WBC, (109/L) | 10.20 [7.10, 14.10] | 10.10 [7.00, 14.00] | 10.40 [7.22, 14.67] | 0.364 |
| N, (109/L) | 4.44 [3.48, 9.31] | 4.30 [3.41, 9.00] | 4.80 [3.88, 9.52] | <0.001 |
| L, (109/L) | 2.84 [2.71, 2.96] | 2.82 [2.70, 2.92] | 2.99 [2.74, 3.23] | <0.001 |
| CRP, (mg/dL) | 11.00 [2.40, 81.31] | 9.60 [2.10, 85.75] | 14.10 [4.00, 55.54] | 0.027 |
| eGFR, mL/min/1.73m2 | 45.08 [29.05, 68.33] | 47.25 [30.85, 68.85] | 36.64 [25.97, 65.46] | 0.003 |
| **Comorbidities** |  |  |  |  |
| Sepsis, n (%) | 187 (22.3) | 121 (19.2) | 66 (31.4) | <0.001 |
| Stroke, n (%) | 39 (4.6) | 27 (4.3) | 12 (5.7) | 0.508 |
| Pneumonia, n (%) | 396 (47.1) | 280 (44.4) | 116 (55.2) | 0.008 |
| AMI, n (%) | 66 (7.9) | 45 (7.1) | 21 (10.0) | 0.236 |
| Liver disease, n (%) | 840 (100.0) | 630 (100.0) | 210 (100.0) | >0.99 |
| COPD, n (%) | 35 (4.2) | 28 (4.4) | 7 (3.3) | 0.618 |
| Cancer, n (%) | 26 (3.1) | 17 (2.7) | 9 (4.3) | 0.357 |
| Hypertension, n (%) | 339 (40.4) | 253 (40.2) | 86 (41.0) | 0.903 |
| DM, n (%) | 320 (38.1) | 243 (38.6) | 77 (36.7) | 0.682 |
| AF, n (%) | 465 (55.4) | 344 (54.6) | 121 (57.6) | 0.496 |
| CKD, n (%) | 327 (38.9) | 244 (38.7) | 83 (39.5) | 0.902 |
| Respiratory failure, n (%) | 383 (45.6) | 257 (40.8) | 126 (60.0) | <0.001 |
| Saps II score | 44.00 [36.00, 53.00] | 42.00 [35.00, 52.00] | 50.00 [42.00, 57.75] | <0.001 |
| Sofa score | 5.00 [3.00, 8.00] | 5.00 [3.00, 7.00] | 6.00 [4.00, 8.00] | <0.001 |
| 24-hour urine output, ml | 1195.00 [676.08, 1945.00] | 1262.13 [718.25, 2038.75] | 1001.50 [516.25, 1657.76] | 0.001 |
| RRT, n (%) | 106 (12.6) | 67 (10.6) | 39 (18.6) | 0.004 |
| Ventilation, n (%) | 744 (88.6) | 552 (87.6) | 192 (91.4) | 0.168 |
| Vasopressin, n (%) | 443 (52.7) | 298 (47.3) | 145 (69.0) | <0.001 |
| Length of ICU stay, days | 4.17 [2.07, 7.76] | 3.62 [1.96, 6.96] | 5.94 [3.47, 11.24] | <0.001 |
| Length of hospital stay, days | 9.94 [6.46, 16.01] | 9.85 [6.46, 15.59] | 10.50 [6.62, 18.38] | 0.043 |
| Platelet counts, 10^9^/L | 202.00 [151.75, 266.00] | 209.00 [160.00, 270.00] | 182.00 [119.00, 251.00] | <0.001 |

Abbreviation: HR, heart rate; RR, respiratory rate; SBP, systolic blood pressure; DBP, diastolic blood pressure; PH, potential of hydrogen; PaO2, partial pressure of oxygen; PaO2, partial pressure of oxygen; PaCO2, partial pressure of carbon dioxide; AMI, acute myocardial infarction; COPD, chronic obstructive pulmonary disease; DM, diabetes mellitus; AF, atrial fibrillation; CKD, chronic kidney diseases; SAPS II, simplified acute physiologic score II; SOFA score, sequential organ failure assessment; RRT, renal replacement therapy; NT-proBNP: N-Terminal pro-brain natriuretic peptide; WBC, white blood cell; N, neutrophile; L, lymphocyte; CRP, C reactive protein, and eGFR, estimated glomerular filtration rate.

**Supplementary Table 3.** Threshold effect analysis of relationship between platelet counts and in-hospital death among critical AHF patients.

| Platelets Inflection Point | OR (95% CIs) | *P*-value |
| --- | --- | --- |
| Fitting by adjusted logistic model | 0.997 (0.994,0.999) | 0.018 |
| Inflection point | 209×10^9^ /L |  |
| ≤209×10^9^ /L | 0.990 (0.985, 0.995) | <0.001 |
| >209×10^9^ /L | 1.002 (0.998, 1.006) | 0.380 |
| Log likelihood ratio test | 0.003 | |

Abbreviations: OR, odds ratio; CI, confidence interval

**Supplementary Table 4.** The evolution of platelet counts between hospital survivors and hospital non survivors After being admitted to the intensive care unit.

| Time | PLT, mean (SD) median (25th-75th percentile) | |  |
| --- | --- | --- | --- |
|  | Survivors | Non-survivors | *P*-value |
| On first day | 218.99(82.67) 213 (164-266) | 196.04 (86.96) 184 (135-250) | <0.001 |
| On 2–3th day | 206.89 (84.27) 201 (148-255) | 185.15 (97.46) 176 (111-240) | <0.001 |
| On 4-5th day | 211.79 (87.13) 206(150-264) | 171.42 (95.04) 162 (95-236) | <0.001 |
| On 6–7th day | 221.48 (90.58) 216 (156-277) | 173.77 (102.57) 161(92-238) | <0.001 |

**Supplementary Table 5.** ROC for PLT, eGFR, and NT-proBNP on predicting in-hospital mortality.

| **Variables** | **AUC** | **SE** | **95% CI** |
| --- | --- | --- | --- |
| PLT | 0.607 | 0.0223 | 0.589 - 0.625 |
| eGFR | 0.681 | 0.0195 | 0.664 - 0.698 |
| NT-proBNP | 0.678 | 0.0200 | 0.660 - 0.695 |
| PLT+NT-proBNP | 0.687 | 0.0206 | 0.670 - 0.704 |
| PLT+eGFR | 0.697 | 0.0197 | 0.680 - 0.713 |

Abbreviations: ROC: receiver operating characteristic; CI, confidence interval; AUC: area under the curve; PLT: platelet; eGFR, estimated glomerular filtration rate; NT-proBNP: N-Terminal pro-brain natriuretic peptide.

**Supplementary Figure 1.** Correlation analysis of in-hospital mortality with WBC, CRP, neutrophil, lymphocyte, and PLT.


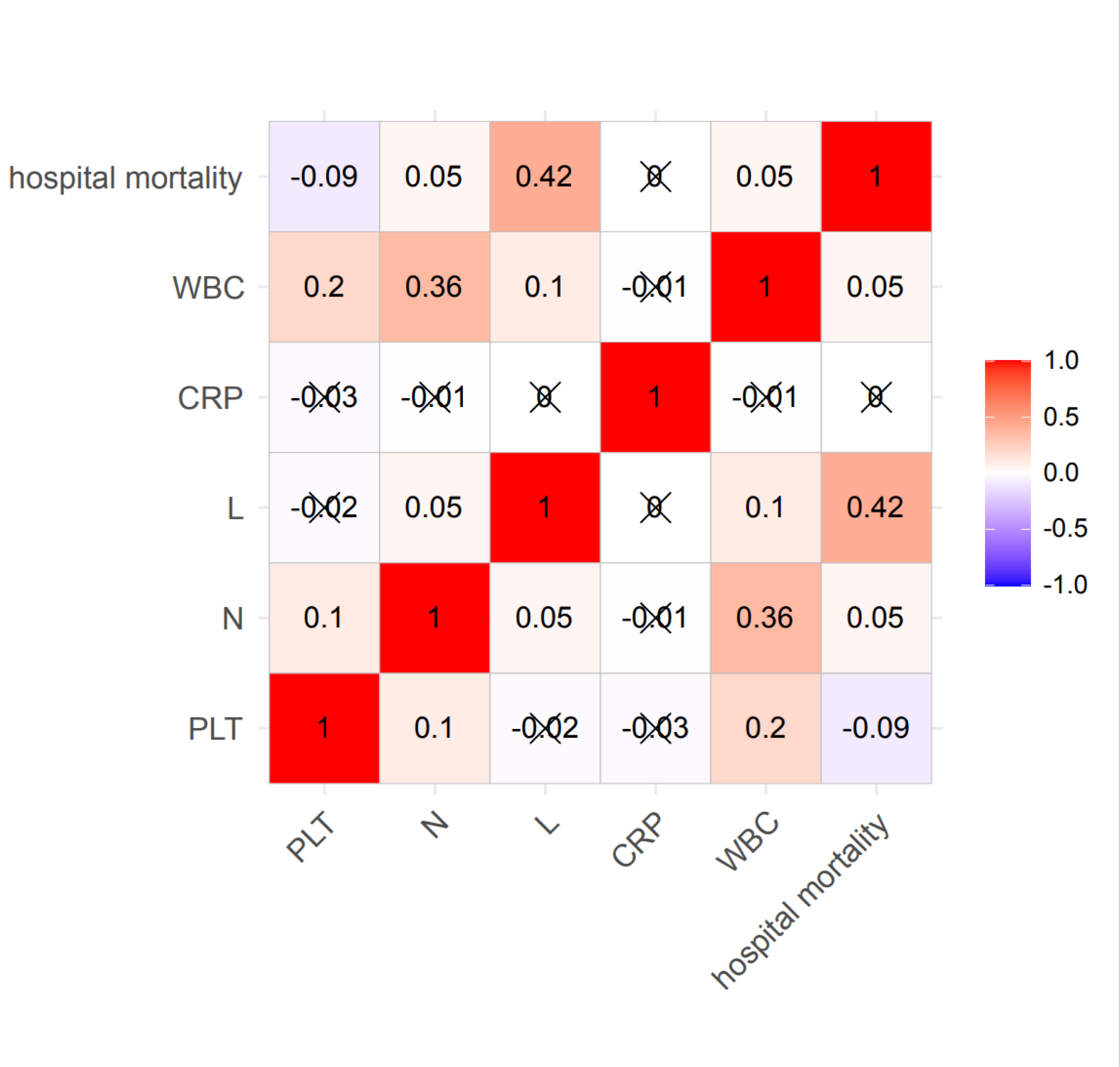


X: *P*-value greater than 0.05.
